# High resolution acoustic telemetry reveals swim speeds and inferred field metabolic rates in juvenile white sharks (*Carcharodon carcharias*)

**DOI:** 10.1371/journal.pone.0268914

**Published:** 2022-06-09

**Authors:** James M. Anderson, Emily Spurgeon, Brian S. Stirling, Jack May, Patrick. T. Rex, Bobby Hyla, Steve McCullough, Marten Thompson, Christopher G. Lowe

**Affiliations:** 1 Department of Biological Sciences, California State University Long Beach, Long Beach, California, United States of America; 2 School of Statistics, University of Minnesota, Minneapolis, Minnesota, United States of America; Institut de Recherche pour le Developpement, FRANCE

## Abstract

White sharks (*Carcharodon carcharias*) are the largest shark species to display regional endothermy. This capability likely facilitates exploitation of resources beyond thermal tolerance thresholds of potential sympatric competitors as well as sustained elevated swim speeds, but results in increased metabolic costs of adults, which has been documented in different studies. Little, however, is known of the metabolic requirements in free-swimming juveniles of the species, due to their large size at birth and challenges in measuring their oxygen consumption rates in captivity. We used trilateration of positional data from high resolution acoustic-telemetry to derive swim speeds from speed-over-ground calculations for eighteen free-swimming individual juvenile white sharks, and subsequently estimate associated mass-specific oxygen consumption rates as a proxy for field routine metabolic rates. Resulting estimates of mass-specific field routine metabolic rates (368 mg O_2_ kg^−1^ h^−1^ ± 27 mg O_2_ kg^−1^ h^−1^ [mean ± S.D.]) are markedly lower than those reported in sub-adult and adult white sharks by previous studies. We argue that median cruising speeds while aggregating at nearshore nursery habitats (0.6 m s^-1^ [mean ± S.E = 0.59 ± 0.001], 0.3 TL s^-1^) are likely a feature of behavioral strategies designed to optimize bioenergetic efficiency, by modulating activity rates in response to environmental temperature profiles to buffer heat loss and maintain homeostasis. Such behavioral strategies more closely resemble those exhibited in ectotherm sharks, than mature conspecifics.

## Introduction

A fundamental component of understanding ecology and evolution in animals is understanding biophysical processes (e.g. metabolism) that allow them to exploit specific resource and habitat niches. Metabolic processes have an associated cost (energy). Thus, energy available for metabolic processes is constrained by the summed energetic requirement of all other essential biological functions and processes.

White sharks (*Carcharodon carcharias*) are the largest fish species to exhibit regional endothermy—the ability to compartmentally regulate internal body temperature relative to the external environment [1]. This capability enables animals to tolerate a broader range of temperatures, and likely optimizes key physiological processes [1,2], but is energetically expensive [3,4]. While it is unclear how endothermic capability specifically varies with ontogeny in white sharks, the surface area to volume ratio of the body is larger in juveniles, and their muscle mass is considerably less, thus they are likely more susceptible to heat loss to the external environment and may rely more on behavioral thermoregulatory strategies [5].

In obligate ram-ventilating fishes, such as white sharks, a key energetic cost is locomotion (swimming). Previous studies in adults and sub-adults of the species have suggested that white sharks have developed behavioral strategies that help offset the energetic costs of regional endothermy [6], including elevated average swim speeds (cruising speeds) [3] in comparison with species that do not exhibit regional endothermy, that provide a competitive advantage in resource exploitation. This hypothesis was supported by the results of a recent study that used a synthesis of bio-logging data from both ectotherm and endotherm fish, and concluded that the convergent evolution of endothermy in a range of fishes more likely driven by competitive advantages gained in ecological interactions and resource exploitation, rather than by thermal niche expansion [7]. Juvenile white sharks (JWS) exploit a very different habitat niche to their adult conspecifics [5,8–14], and rely on a mostly piscivorous based diet [15–19]. They also form loose aggregations using relatively small areas (< 4 km^2^) along coastal beaches spending days to months in these nursery habitats where water is warmer, prey species are more abundant and there are few larger predators [20,21]. Thus, understanding swim speeds and associated metabolic costs in JWS allows inference of their energetic requirements, and therefore carrying capacity of these nursery areas. Generally, smaller fish species, or smaller ontogenetic stages of a species can be used in captive empirical studies to measure oxygen consumption rates against manipulated variables (e.g ambient temperature, swim speed, body size) as a proxy for metabolic costs [22–27]. However, larger bodied fish, such as white sharks, are more challenging to keep in captivity, and measure oxygen consumption rates in flumes or swim tunnels. Thus, attempts to assess energetic expenditure may be more feasible by estimating oxygen consumption rates in free-swimming animals (field metabolic rates). A commonly used approach is metabolic scaling, whereby the allometric relationship between body size and metabolic rate is described and accounted for by a scaling exponent in a linear regression equation derived largely from animals swum in flumes/swim-tunnel respirometers at ‘preferred’ swimming speeds [28]. A limitation of this approach is that datapoints from which the slope applied to this equation are derived are ‘bookended’ by smaller animals, leading to a likelihood that error is introduced when extrapolating to large animals [28]. An alternative approach to estimating energetics of free-swimming white sharks was described by Semmens *et al*. (2013) [29], who used a linear regression that modified a previously described [30] relationship between metabolic rates and swimming speeds (see methods). This approach allows for characterization of routine energetic costs associated with specific behavioral suites but may also yield over-estimates if regarded as the routine metabolic rate across all behavioral suites.

**Fig 1:**
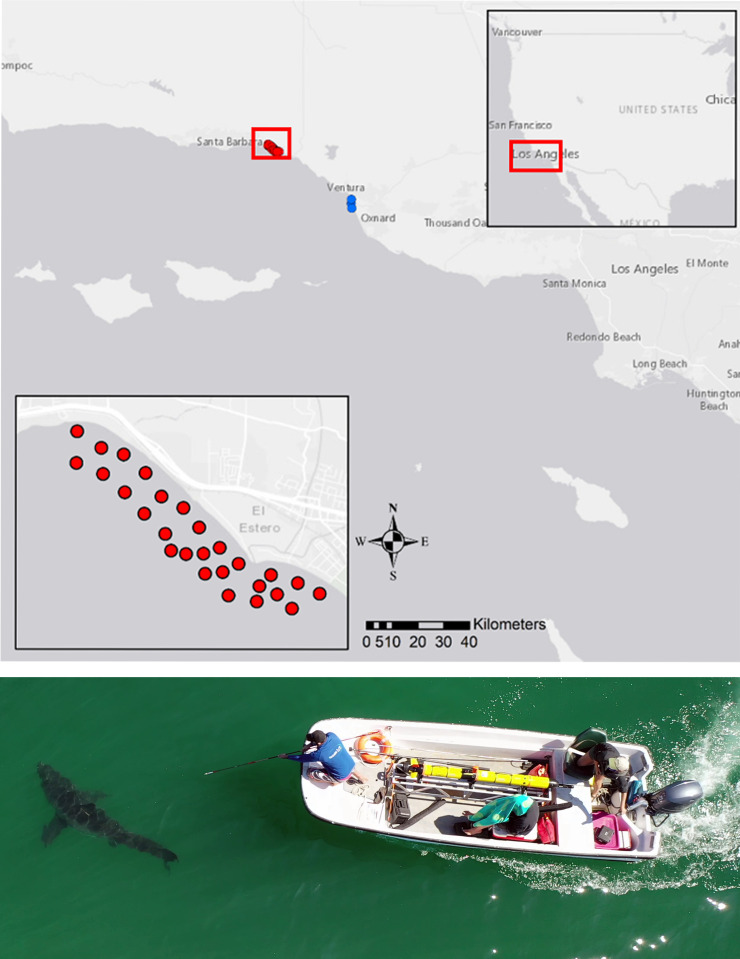
White shark tagging and study location. Main map (top) shows the location of the VPS array (red dots) just North of Carpinteria, CA, USA, as well as three acoustic monitoring locations off Ventura, CA, USA (blue dots). Inset map shows area magnified view of the study area and VPS array. Basemap and map data were produced in ArcGIS software by ESRI, using map imagery available from USGS (https://apps.nationalmap.gov/viewer/). Image (bottom) shows a juvenile white shark being tagged within the VPS array.

Here, we use high-resolution acoustic telemetry positioning data derived via hyperbolic trilateration [31] from tagged JWS detected within a Vemco Positioning System (VPS) array (Fig 1) to determine horizontal swim speeds (SS_*H*_), based on speed-over-ground measurements. Calculated average swim speeds (cruising speeds) within the array, and when in transit between geographic locations, revealed average swim speeds are lower than those previously reported in adults and sub-adults and are associated with behavioral mode switching (residency vs migration). We estimate and compare field routine metabolic rates (fRMR) for these sharks by following the methods of Semmens *et* al (2013) and Payne *et* al (2015). These findings have important bearing upon daily energetic rates, particularly in fast growing juvenile white sharks. In addition, this large activity dataset provides essential information for modelling energetic requirements, and thus environmental carry capacity, as well as movement modelling processes (such as state space models and hidden Markov models) that rely on more accurate estimates of movement rates.

## Results

### Speed over ground & true velocity calculations

Horizontal swim speed (SS_*H*_) estimates were derived from VPS geolocation estimate data of eighteen juvenile white sharks, ranging in estimated size from 152 cm to 315 cm total length (TL; Table 1). As SS_*H*_ calculations for one shark (2020–37) were anomalously low, and were derived from only 5 relocations, data from this shark were removed from subsequent analyses. Filtered data were composed of 58,602 geolocations, from which 58,584 calculations of SS_*H*_ (swim speed) were derived (56,657 used after speeds < 0.1 m s^-1^ removed from the analyses). Calculated SS_*H*_ and SS_*T*_ ranged from 0.1 m s^-1^ to 7.7 m s^-1^. As SS_*H*_ distributions showed a strongly positive skew, we present median swimming speeds in these analyses.

**Table 1:** Details of VPS derived cruise speed data for all juvenile white sharks.

Shark ID	TL (cm)	FL (cm)	Mass (kg)	Days Tracked	# Relocations	Median SS_*H*_ (m s^-1^)		S.E	Median SS_*T*_ (m s^-1^)	S.E
2020–13	152	138	32.1	64	3943	0.62	0.004		NA	NA
2020–15	217	196	100.3	110	5285	0.62	0.003		NA	NA
2020–16	315	283.5	332.8	113	5508	0.71	0.004		NA	NA
2020–17	259	233.1	176.2	99	6200	0.66	0.003		NA	NA
2020–19	259	233.1	176.2	107	7802	0.58	0.003		0.58	0.003
2020–20	155	139.5	33.2	107	14106	0.58	0.002		0.58	0.002
2020–21	155	139.5	33.2	107	11256	0.62	0.002		0.62	0.002
2020–22	183	164.7	57.0	102	4167	0.59	0.003		NA	NA
2020–31	243	218.7	143.2	35	728	0.71	0.007		0.71	0.007
2020–32	270	243	201.7	49	7854	0.64	0.004		0.64	0.004
2020–33	183	164.7	57.0	36	3475	0.56	0.008		0.56	0.008
2020–34	239	215.1	135.7	17	1325	0.68	0.005		NA	NA
2020–35	183	164.7	57.0	2	118	0.61	0.018		NA	NA
2020–36	183	164.7	57.0	36	3161	0.56	0.005		NA	NA
2020–40	244	219.6	145.1	8	326	0.62	0.012		NA	NA
2020–41	198	178.2	73.6	2	102	0.60	0.023		NA	NA
2020–42	200	180	76.0	1	34	0.68	0.042		NA	NA

Median SS_*H*_ (cruising speed) across all animals was 0.6 m s^-1^ (mean ± S.E. = 0.6 ± 0.001). Individual median cruising speeds ranged from 0.6 m s^-1^ (0.56 ± 0.008) to 0.7 m s^-1^ (0.71 ± 0.007). Standardized to body size, cruising speed across all tagged animals was 0.3 U TL s^-1^ (0.30 ± 0.001). True swim speeds (SS_*T*_) were calculated for the six animals fitted with pressure (depth) sensing tags. Calculated SS_*T*_ did not differ from calculated SS_*H*_ (block-bootstrap test between medians, p > 0.9898, S1 Fig), indicating that although SS_*T*_ accounts for Δ(depth), and therefore more accurate estimates of distance between derived locations, SS_*H*_ calculations remain valid.

Median SS_*T*_ across all animals included was 0.6 m s^-1^ (0.58 ± 0.001), slightly higher than SS_*H*_. Individual median SS_*T*_’s ranged from 0.6 m s^-1^ (0.54 ± 0.01) to 0.7 m s^-1^ (0.69 ± 0.01) (Table 1). Cruising speed calculations derived from unmanned aerial vehicle (UAV) tracking of 25 individual JWS swimming within the array, in a single day, were also marginally lower than those of calculated SS_*H*_ when standardized to body size. Median calculated velocity via UAV was 0.6 m s^-1^ (0.66 ± 0.005). Corresponding median cruising speed, standardized to body size were 0.3 U TL s^-^1 (0.29 ± 0.002), slightly lower than those derived via calculated SS_*H*_ (Table 2).

**Table 2:** UAV derived JWS cruise speeds.

Shark ID	Size (TL cm)	# Observations	Median SwimSpeed (TL s^-1^)
1	290	29	0.29
2	215	41	0.33
3	244	62	0.31
4	234	44	0.27
5	242	39	0.29
6	221	83	0.24
7	235	47	0.29
8	240	48	0.27
9	216	57	0.27
10	219	74	0.30
11	242	43	0.26
12	230	57	0.27
13	260	44	0.31
14	224	48	0.31
15	260	48	0.29
16	209	51	0.25
17	209	46	0.37
18	260	43	0.24
19	247	39	0.29
20	187	39	0.38
21	191	62	0.26
22	251	45	0.24
23	237	50	0.26
24	182	51	0.27
25	230	42	0.29

### Relationship of cruising swimming speed to biotic and abiotic variables

Water temperature within the array was observed to be largely stratified by depth, with temperatures ranging from 12.5°C to 22.4°C (mode = 15.4°C, mean = 18.1°C ± 0.01°C, median = 17.8°C. Six percent of all rendered geopositions were associated with corresponding water temperatures of 15°C or less, while 55% corresponded to temperatures between 15°C and 18°C.

We compared calculated swim speeds against a range of biotic and abiotic variables. The nature of this telemetry data likely induced autocorrelation, and we employed tools that addressed this. Namely, we performed hypothesis tests using a block bootstrap approach that is robust to serial correlation. Table 3 contains a summary of our results. As mentioned above, we used this method to test whether median SS_*H*_ *differed* from SS_*T*_; it was not significant (p > 0. 9898). Additionally, we tested whether median cruising speeds in sharks was the same between female, male, and unidentified sharks. Female sharks were significantly faster than male and unidentified sharks, and male sharks did not differ significantly from unidentified sharks. In order to maintain a familywise error rate of α = 0.05 across the three tests concerning sex, we used a Bonferroni corrected significance level of α = 0.05/3 = 0.0167 for these tests (i.e., $SS_{H}(F)=SS_{H}(M),SS_{H}(F)=SS_{H}(U),SS_{H}(M)=SS_{H}(U)$). This observation may be a function of sample size difference by sex (female = 9, male = 2, unidentified = 6: S1 Fig). Further details pertaining to the block bootstrap and our implementation may be found in the Methods section.

**Table 3:** Hypothesis tests for typical swim states.

H_*0*_		p-value
SS_*H*_ = SS_*T*_		> 0. 9898
SS_*H*_ (Day) = SS_*H*_ (Night)		< 0.0001
SS_*H*_ (F) = SS_*H*_ (M)		< 0.0167
SS_*H*_ (F) = SS_*H*_ (U)		< 0.0167
SS_*H*_ (M) = SS_*H*_ (U)		< 0.0167

**Fig 2:**
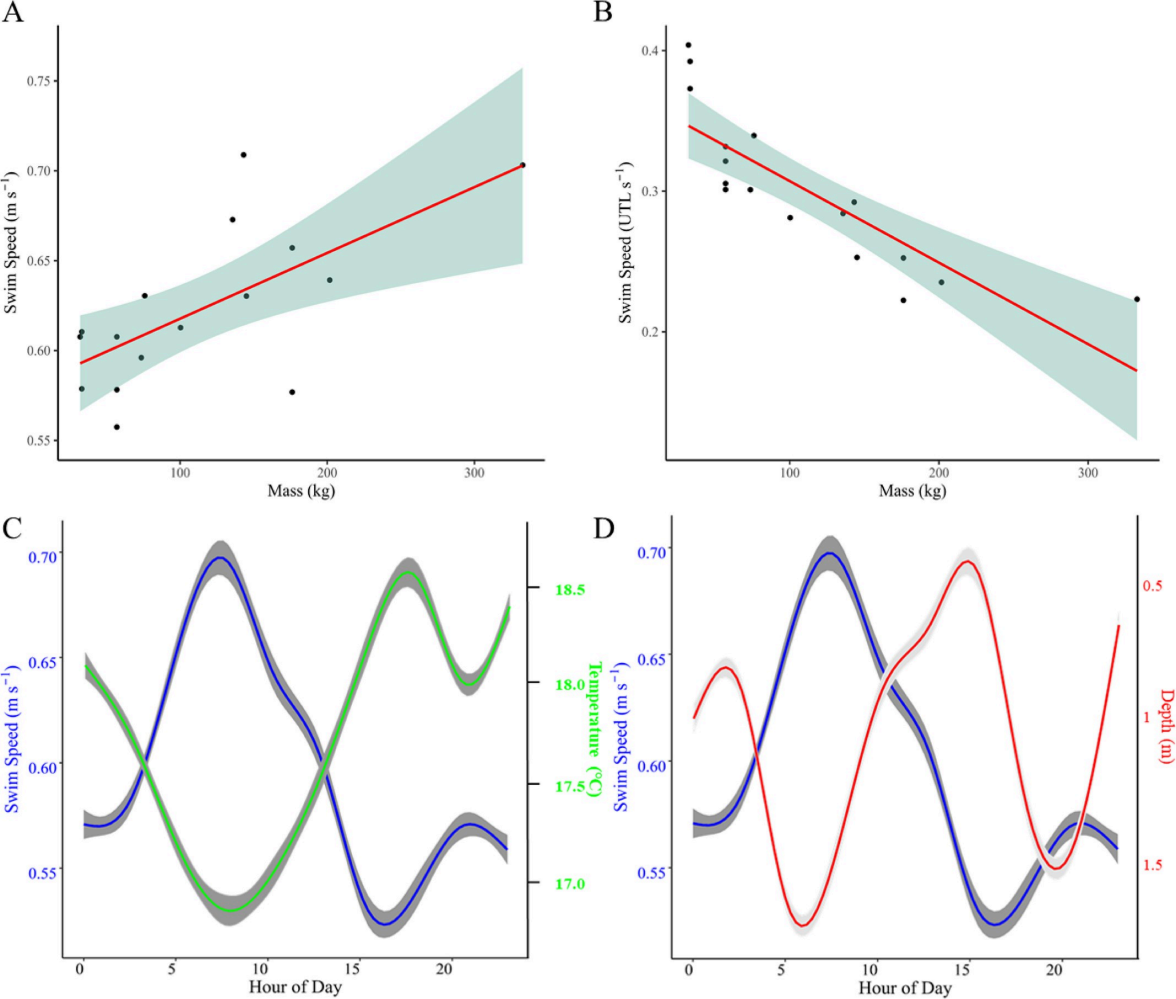
Relationship of cruising speed to shark size, diel period, temperature and depth. **(A)** Median values of raw cruising speeds (m s^-1^) for each shark were found to have a weak positive linear relationship to shark body size, described by the equation (SS_H_ = 5.3x10^-5^(M) + 0.61). Data points show the mass (kg) and cruising speed (m s^-1^) of each shark included in the study. **(B)** Standardized to total length, a negative correlation between median cruising speed and body mass (size) was observed, with larger sharks effectively swimming more slowly than smaller sharks. Data points show the mass (kg) and cruising speed standardized to the total length of the animal (UTL s^-1^) for each shark included in the study. **(C)** Relationship between cruising speed (m s^-1^, blue trace) and environmental temperature (green trace°C) for the six sharks equipped with depth sensing tags. Trend line shows conditional smoothed mean values, grey shading indicates 95% confidence intervals. **(D)** Relationship between cruising speed and depth for the same six sharks. Blue trace indicates mean swim speed (m s^-1^), red trend line indicates mean swimming depth (m).

Calculated cruising speeds (median SS_*H*_) were positively correlated with shark size (Pearson’s product-moment correlation, r = 0.27, Fig 2A). Standardized to body size (TL cm), cruising speeds were negatively correlated with body size (Pearson’s product-moment correlation, r = -0.83, Fig 2B). Cruising speeds were negatively correlated with water temperature (Pearson’s product-moment correlation, r = -0.12, Fig 2C) and positively correlated with swimming depth (Pearson’s product-moment correlation, r = 0.16, Fig 2D). Due to the possible auto-correlated nature of such data, we do not report on the significance of these specific correlations, providing their values for illustrative purposes only.

**Fig 3:**
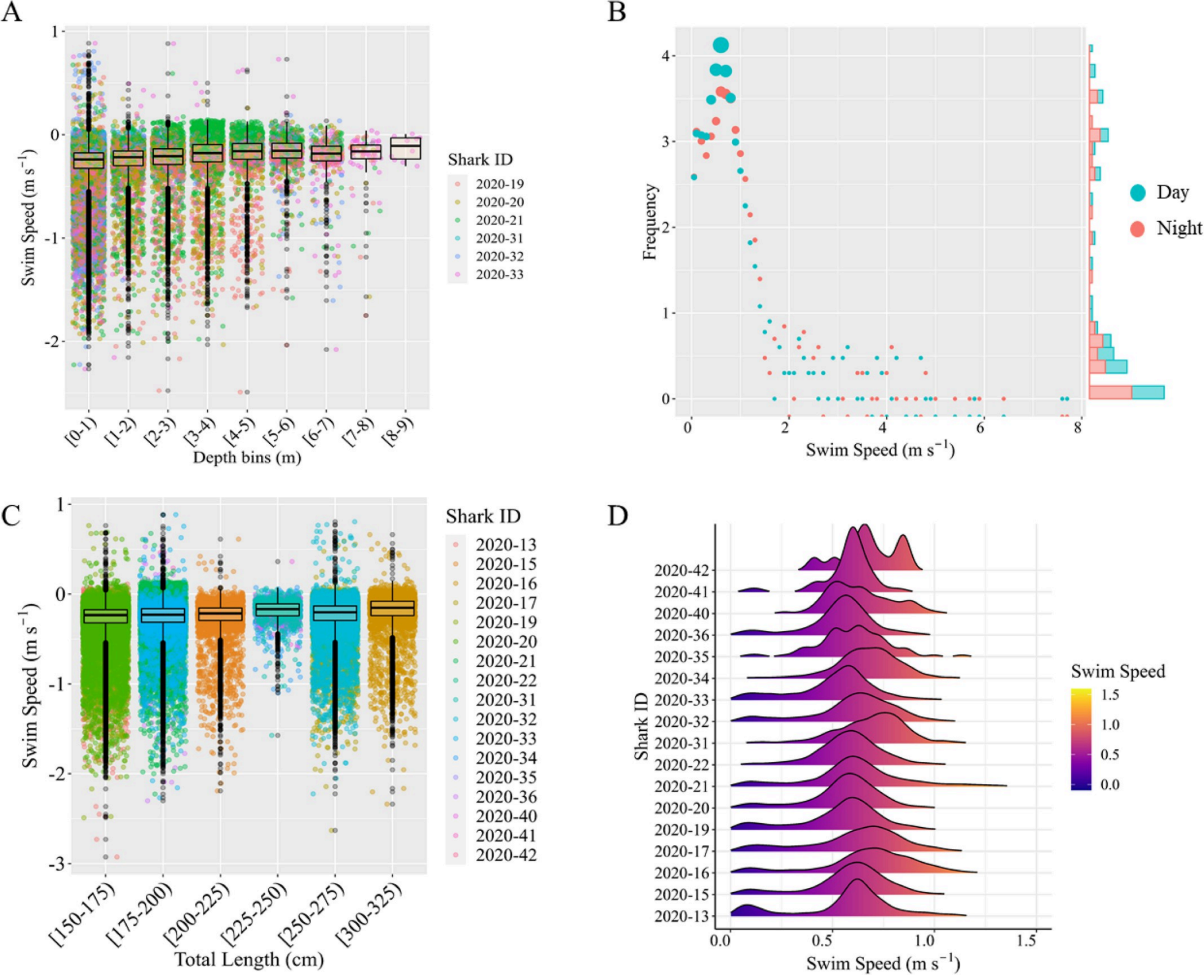
Relationship of juvenile white shark cruising speed to biotic and abiotic variables. **(A)** Box and whisker plot of swim speeds (in m s^-1^, log transformed) associated with depth bins (in meters). A great majority of locations (and thus calculated velocities) occurred within depths from 1–5 meters. No difference was seen in median SS_*T*_ with changes in depth profile. **(B)** Frequency distribution and marginal histogram plot of diel patterns in JWS cruising speeds. Dot size is relative to frequency (log transformed). Significantly more derived locations occurred by night. SS_*H*_ patterns at night exhibited a significantly larger range, as well as significantly higher maximum values compared with daytime patterns. Marginal histogram shows distributions of plotted variables. **(C)** Box and whisker plot of swim speeds (in m s^-1^, log transformed) associated with shark size-class. **(D)** Ridgeline plot of SS_*H*_ distributions by individual shark (see Table 1 for reference).

A general trend of higher median swim speeds across binned depth ranges was observed (Fig 3A). We do not report statistical results here due to inherent autocorrelation. Although raw SS_*H*_ values at night were greater than those during the day (Fig 3B), median SS_*H*_ was actually greater by day than by night (0.64 m s^-1^ vs 0.59 m s^-1^), night-time swim speeds were 0.04m/s slower than daytime swim speeds (batch-bootstrap test, p < 0.0001: Fig 3B). No difference was seen in median SS_*H*_ when tested against size (Fig 3C).

### Median velocity under transitory behaviors

Approximate SS_*H*_ values associated with transit (SS_*H*_*Transit*) between the VPS array and a small acoustic receiver array to the South (off Ventura, CA) were calculated for 12 individual sharks, using the elapsed time and the Vincenty distance between the last detection on the VPS array and the first detection at monitored sites off Ventura (see Fig 1 for reference). Mean distance between relocations (VPS array (Carpinteria) to Ventura array) was 28.78 km ± 1.3 km (mean ± S.D.). Individual transit times between the two locations ranged from 2 hrs 40 mins to 22 hrs 23 min. Median transit time was 9 hrs 47 min ± 36 min. Transit times > median transit time were excluded from SS_*H*_*Transit* calculations. Thus, final SS_*H*_*Transit* was calculated from 9 individuals. Mean calculated SS_*H*_*Transit* was 0.981 m s^-1^ ± 0.717 (mean ± S.D.).

### Estimated field routine metabolic rates

**Fig 4:**
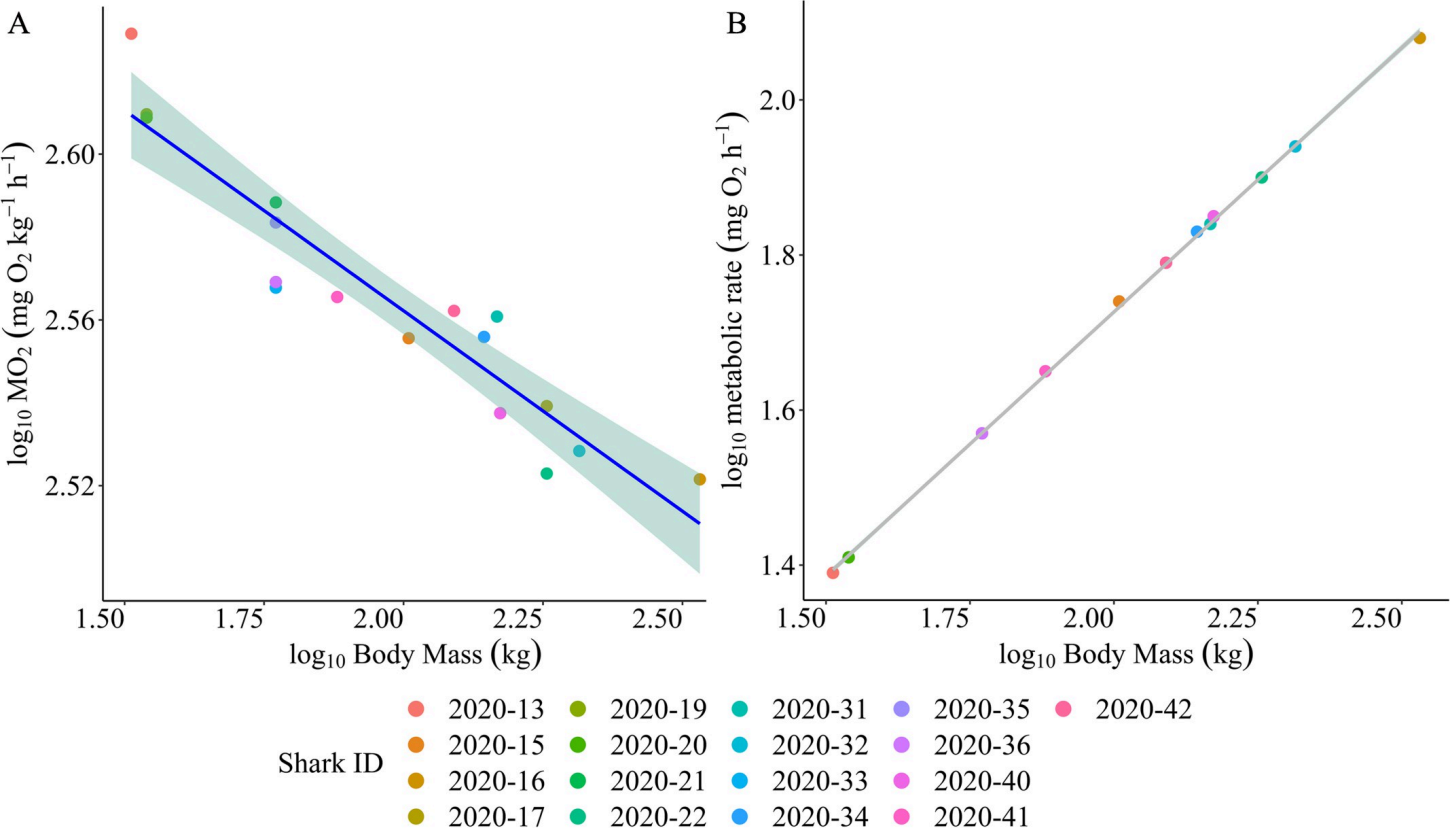
Metabolic implications of measured swimming speeds and body size in juvenile white sharks. **(A)** Estimated mass-specific mean fRMR of juvenile white sharks in the study. Points and trend-lines show mean estimated oxygen consumption rates of all sharks included in analyses, with respect to body size (mass), based on the general equation furnished by Semmens et al. (2013). Shaded areas delineate 95% confidence intervals associated with conditional smoothed mean values. **(B)** Whole-body routine metabolic rates (mean 420 ± 51mg O_2_ h^−1^) based upon the allometric mass-scaling exponent of 0.79, as described by Payne et al (2015).

We calculated field routine oxygen consumption rates (fRMR [MO_2_]) and whole body RMR of all JWS resident within the array that were used in this analysis, following methodologies described by Semmens et al. (2013) [29], and Payne et al. (2015) [28] respectively. Modal and median water temperature selected by sharks equipped with depth sensing tags within the array was within the range of mean water temperatures reported by Ezcurra et al. (2012 [15.2°C to 17.9°C]) [26] in their calculations of routine metabolic rates of captive young of the year white sharks. These temperature results were subsequently incorporated into the Semmens et al. (2013) model for estimating fRMR. Thus, we did not correct for temperature in our estimates of fRMR. Following the methodology put forward by Semmens et al (2013) and based upon the median cruising speed of 0.3 TL s^-^1, the mean field routine metabolic rate of JWS displaying resident behaviors within the array was estimated to be 368 mg O_2_ kg^−1^ h^−1^. Estimated oxygen consumption rates associated with median cruising speeds in individual sharks ranged from 332 to 426 mg O_2_ kg^−1^ h^−1^, with a mean mass-specific (± S.D) value across all sharks of 362 ± 39 mg O_2_ kg^−1^ h^−1^ (Fig 4A). By comparison, estimated whole body routine metabolic rates calculated via the methodology described by Payne et al. (2015) ranged from 341 to 512 mg O_2_ h^−1^ (Fig 4B), with a mean of 420 ± 51 mg O_2_ h^−1^. Based upon the mean shark body-mass (111.02 kg, 214 cm [TL]), and mean SS_*H*_*Transit* (0.98 m s^-1^, 0.46 TL s^-1^), we estimated fRMR associated with transit behavior to be in the order of 454 mg O_2_ kg^−1^ h^−1^.

## Discussion

### Juvenile white shark cruising speeds and routine metabolic rate estimation

Our calculations of cruising speeds (SS_*H*_; 0.6 m s^-1^, 0.3 TL s^-1^) in acoustically tagged JWS displaying resident behaviors are close to net velocities reported in a recent study [32] that derived swim speeds of JWS tracked by a UAV (0.61 m s^-1^), and are distinctly lower than many published average swim speeds for adult and sub-adult conspecifics [4,15,29,33]. The slow cruising speeds of JWS in this study, exhibiting resident behaviors at nursery locations, suggests they may adopt a fundamentally different behavioral strategy, whereby they may seek to behaviorally maximize bioenergetic efficiency [34,35] by minimizing energetic costs associated with locomotion. A weak-positive relationship between body size and raw cruising speed was observed. This observation is similar to that described across a range of shark species [36], and is likely a simple function of kinematics and caudal fin morphology across different sized JWS. However, when standardized against body length (in TL s^-1^) cruising speeds in JWS included in the study were observed to follow a negative relationship with shark body size, whereby smaller white sharks, with smaller bodies, exhibited higher swim speeds than those of larger conspecifics. Harding et al., (2021) [7] found modal swimming speeds of sub-adult and adult white sharks (320–430 cm TL; n = 5) to range between 0.8–0.98 m s^-1^. Standardized to body length, these equate to modal speeds of 0.21–0.25 m s^-1^, with the smaller sharks exhibiting higher modal swimming speeds than larger sharks.

While the accuracy of our size estimations is subject to variability, the relationship of swim speed and shark size was not well explained by size alone (R^2^ = 0.071), indicating there are other contributing factors that likely influence cruising speeds in juvenile white sharks. Although gait patterns in movement behaviors have been demonstrated to be similar in a range of marine vertebrates, including sharks [6], the relationship between size and energetic efficiency is argued to be best described by distinct allometric scalings for large and small swimmers [37]. Unfortunately, this relationship has yet to be described in white sharks. Therefore, while cruising speed in sharks can be well explained by the combination of shark size (fork length) and caudal fin morphology [36], cruising speeds in JWS in this study might be better explained by body mass, body temperature and endothermic capacity [3].

Routine metabolic rate (RMR) refers to the average metabolic rate of an animal undergoing normal behaviors [38], and accounts for standard metabolic rate, body temperature and the metabolic cost of swimming [39]. If we consider swim speed (a proxy for activity rate) to be directly proportional to metabolic rate [29,35], for the average shark body size across our sample (111 kg, 214 cm [TL]), cruising speed and estimated fRMR were 0.3 U TL s^-1^ (0.6 m s^-1^) and 368 mg O_2_ kg^−1^ h^−1^ respectively, based upon the application of the Semmens *et al*. (2013) approach.

Comparison of our calculated cruising speeds with speed-over-ground measurements derived from UAVs [this study, Colefax et at. (2020) [32]] demonstrates that despite potential error in our spatial resolution (5 m) conferring a greater chance of under-estimation in our calculated cruising speeds, our calculated speeds were in-fact more conservative. Estimation of whole-body routine metabolic rates per the methods described by Payne et al. (2015) yields higher estimated mean oxygen consumption rates overall compared with our swim-speed derived field routine metabolic rate estimates (420 ± 51 mg O_2_ h^−1^ vs 368 ± 39 mg O_2_ kg^−1^ h^−1^ respectively). This observation is likely a function of over-estimation of routine metabolic rate in larger fishes via the body size to metabolic rate regression, as regression slopes and allometric scaling exponents are derived from studies that are largely book-ended by smaller bodied subjects. There is considerable debate as to whether there can be a universal allometric metabolic relationship across species, within species, or even across ontogeny [22,40–44]. A recent study found strong evidence for an applicable universal scaling exponent of 0.89 across fishes [45]. However, in this context, we applied an allometric scaling exponent of 0.79 in our whole body metabolic rate estimation, as it is the exponent that has been incorporated into previous estimates of routine metabolic rates within the species^26, 28^, and represents the best available and most applicable information. Similarly, changing the coefficient applied to the equation that describes the relationship between estimated oxygen consumption rate and swim speed [e.g., Watanabe et al. (2019), Semmens et al. (2013)], which factors the allometric relationship between oxygen consumption rates and body size, changes the magnitude of those oxygen consumption estimates (S2 Fig).

### Behavioral adaptations to endothermic inefficiency

Endothermic capability is argued to facilitate increased cruising speeds, but comes at the cost of increased metabolic demand (increased O_2_ consumption rates) [4,29]. This in turn, along with behavioral adaptations to maximize swim efficiency in a three dimensional environment [6,46], is argued to underpin the success of hunting strategies employed by larger white sharks [4]. Our estimate of cruising speed (0.6 m s^-1^, 0.3 U TL s^-1^) is markedly lower than that recorded by both Carey et al. (1982 [0.89 m s^-1^, 0.51 TL s^-1^], shark size 457 cm TL, 943 kg) [33], more than half that of Semmens et al. (2013) [2.25 m s^-1^, 0.62 TL s^-1^, shark sizes 280–450 cm TL, 195–839 kg] [29], and ~ 2/3 the mean swim speed reported by Watanabe et al. (2019) [0.94 m s^-1^, shark sizes 290–420 cm TL, 218–721 kg] In fact, our cruising speed estimates are more in line with those reported from biologging data by Harding et al., (2021) [7]. Our mean estimated fRMR was approximately half that reported by Semmens et al. (723 mg O_2_ kg^−1^ h^−1^) [29]. These stark differences likely reflect differences in behavioral strategies of white sharks at the sizes/ontogenetic stages of the fish included in the study, as well as possible overestimation of swimming speeds in some studies. Field RMR calculations presented by Semmens et al. (2013) [29] were derived from adults utilizing foraging/hunting habitat (a pinniped colony), thus, reflect different horizontal and vertical behaviors to the animals in the present study. Sharks in the present study equipped with pressure (depth) sensing tags (n = 6) exhibited a selective preference for relatively warmer waters (6% of rendered geolocations associated with water temperatures of 15°C or less), and exhibited behaviors that reflect crepuscular foraging, with peak cruising speeds and deepest swim depths in and around dawn, which likely reflects active hunting, but also corresponds to the being within colder waters. Elevated cruising speeds seen at these times may serve to increase the chance of encountering prey^4,30^ (for example speed of locomotion governs the rate at which an image moves across the retina [47]), and to generate heat during sustained activity periods within colder (deeper) climes. Conversely, minimum cruising speeds were associated with shallow and surface swimming in warmer waters, in the mid to late afternoon, which may facilitate insolation and active warming of the body, whereby sharks may minimize movement rates to maximize energy gains through behavioral thermoregulation [39]. This was observed to be followed by a less pronounced increase in cruising speed associated with deeper swim depths in the hours around sunset. These behavioral patterns reflect those of broadly similar sized white sharks reported by Colefax et al (2020) [32]. Predator-prey interaction models suggest that predator foraging rates should be optimized to when the chances of success are highest, and that foraging success is influenced by biotic factors (for example physiology) and abiotic factors (e.g. ambient temperature) [48,49]. Thus, we may expect JWS that prey upon largely poikilothermic species to be most active when prey species movements are physiologically constrained by diel changes ambient temperature [48].

Increased body temperature of endothermic fishes facilitates more cost-efficient swimming via reduced muscle fiber activation [50]. Standardized to body length, smaller JWS were found to exhibit faster cruising speeds than their larger conspecifics. This observation may be a function reduced thermal efficiency in smaller bodied JWS. Red muscle volume in ectotherm and endotherm sharks, including white sharks, is isometric to body size [51,52]. While trunk red muscle (which generates heat) grows at a constant proportion to somatic growth, white muscle contribution to overall somatic growth is allometric [51,52]. Thus, the proportion of red muscle that contributes to total body mass is greatest as neonates and declines as the animal grows, and the proportional contribution of white muscle to body mass increases. Smaller juvenile white sharks therefore generate more heat per unit mass than their larger counterparts. In fact, mass-specific heat production from trunk-red-muscle in neonates is approximately twice that of adults [52]. However, the smaller body size, and larger surface area to volume ratio of smaller white sharks compared to their adult and sub-adult counterparts, likely results in lower thermal inertia, greater heat loss, and reduced endothermic capacity [53]. Limited capacity for regional endothermy in juvenile white sharks may therefore substantially increase metabolic costs (e.g. through having to increase activity rates to maintain temperature), unless they are able to conserve energy, e.g. through behavioral thermoregulation via selection of thermally optimal habitat [34].

Our swim speed data and associated estimates of metabolic rates suggest that juvenile white sharks in the study exhibited a different form of thermoregulatory behavior compared to adults that can exploit significantly greater thermal inertia. There may be a tendency toward the prevalence of such behaviors declining across ontogeny, as juvenile white shark thermal inertia, and thermoregulatory capability develops. The extent and rate of any such behavioral decline remains unknown. Pacific bluefin tuna (*Thunnus orientalis*) exhibit higher minimum metabolic rates than tropical-water associated yellowfin tuna (*Thunnus* albacares). This observation has been suggested to be related to elevated cardiac capacity in Pacific bluefin, driven by thermal-niche expansion [54], although the thermal-niche expansion hypothesis to explain regional endothermy in fishes was recently refuted [7]. It is possible that JWS that reside in warmer nursery habitat have reduced cardiac capacity compared with their sub-adult and adult conspecifics, and that this capacity (and subsequent oxygen demand) increases across ontogeny in line with spatial and thermal ranges. Many ectothermic teleosts make physiological compensatory adjustments to colder temperatures over time, including increasing the volume of intermediate (fast oxidative-glycolytic) muscle fibers, and increasing mitochondrial density in key areas (e.g. red muscle fibers, brain, liver, gill tissue) [55]. Such intermediate muscles have been observed in white sharks [52], but their relative proportions in relation to body mass have not been described. If juvenile white sharks adapt to colder temperatures across ontogeny in similar ways as that in teleost fishes, we could expect a marked increase in O_2_ requirement associated with increased myoglobin and mitochondria.

### Swim-speeds and behavioral-mode switching

Our VPS array was situated within the core area of an established primary nursery habitat. Thus, calculated cruising speeds were derived from a loose aggregation of juvenile white sharks exhibiting highly resident behaviors. In contrast, transitory behaviors associated with relocation to another, separate, habitat resource are usually associated with highly directional movements [4,58]. Although the SS_*H*_*Transit* calculations we made can only be accepted with substantial caveats, due to not knowing the exact path taken by the animals included, they nonetheless demonstrate significantly increased swim speeds (*ergo* metabolic cost) to those associated with resident behaviors. The higher variance seen in estimated SS_*H*_*Transit* among sharks is likely attributed to the lack of resolution using speed-over-ground methods for estimated swimming speeds during transitory movements; however, it may also indicate individual differences in movement behavior. While there is an increased metabolic cost associated with these inter-area transits, they are likely offset by temporal benefits of locating new areas with suitable environmental conditions and greater prey densities. Difference in cruising speeds associated with behavioral mode may also relate to external temperature, as water temperatures encountered during transit will likely differ from those that sharks preferentially selected within a suitable nursery area [10]. Our findings thus lend support to previous observations that juvenile white sharks likely exhibit behavioral patterns of temporary residency and travelling [56], moving between, and stopping at, discrete resource patches.

### Future directions

While the calculated cruising speeds presented here are robust, they are derived from estimated body lengths, as well as a fixed degree of marginal error associated with hyperbolic position estimates [31]. Much of the variation associated with our fRMR and whole-body RMR calculations can likely be attributed to inaccuracy of body length estimates. Future approaches should seek to incorporate multi-sensor packages (e.g., tri-axial magnetometer-accelerometers) with active tracking to derive the highest possible resolution of three-dimensional movement over time. This, combined with physical measurement of body length, rather than estimation would serve to provide the most accurate estimates of true velocity, and associated fRMR Thus, it may be possible to identify size class or ontogenetic stage at which juvenile white sharks’ thermoregulation strategies transition to those employed by larger conspecifics. Such data could also facilitate accurate estimates of absolute energetic requirements, which combined with approaches such as stable isotope analysis, would allow characterization of the carrying capacity of specific habitat resources, such the nursery habitat used by juvenile white sharks in this study.

## Methods

### Ethics statement

All capture and tagging procedures were carried out in accordance with State and federal permits. All experimental protocols were approved under the California State University Long Beach Institutional Animal Care and Use Committee (IACUC); protocol #364.

Juvenile white sharks aggregating within a southern California nursery habitat location were externally tagged with either a Vemco V16 or V13 (Vemco | Innovasea, Nova Scotia, Canada) coded acoustic transmitter (transmitter family V13-1x-069k, V13-2x-069k, V13P-1x-069k-3-0034m, V16-4x-069k, V16-5x-069k. V16-6x-069k). Shark total length (TL cm) was estimated at the time of tagging, and in subsequent review of drone video footage. Length to body mass relationships established by Logan et al. (2018) [57] were used to determine fork length (FL cm) and shark body mass (M kg).

To track the movements and positions of tagged sharks, a VPS acoustic array was deployed off Carpinteria, CA, USA. VPS allows for the use of a time-difference-of-arrival algorithm across three or more receiver stations (trilateration) to determine fine scale locations of an acoustic tag [31,58]. The array was composed of 24 receivers (16 VR2tx, 8 VR2W with attached reference tag), stretching a linear distance of ~2.8 km, with a coverage area of ~ 8 km^2^ (Fig 1). All detection data from receivers in the array were given to Vemco | Innovasea, (Nova Scotia, Canada) to derive shark positions via their hyperbolic positioning algorithm [31], for a total of eighteen individual sharks. Raw derived position data were filtered by their associated horizontal position error estimate (HPE), to include only data where HPE was ≤ 5 (m).

### Shark velocity (speed over ground) calculation

The time elapsed between successive VPS derived locations for each shark was calculated, as was the Vincenty distance between each successive geo-position. Shark speed over ground (hereafter Horizontal swimspeed [SS_*H*_]) was calculated as the Vincenty distance divided by the elapsed time. Having calculated SS_*H*_, all rows of data where the elapsed time was greater than 300 sec. were removed. True swimspeed (SS_*T*_) was subsequently calculated for the six animals outfitted with pressure (depth) sensing tags. True swimspeed accounts for any change in depth between each successive derived position. Calculations assume a constant inclination or declination between two successive VPS geolocation positions, thereby accounting for the hypotenuse of dive angles between points. Thus, SS_*T*_ represents a more accurate calculation of total distance travelled between successive positions.

Swims speeds for JWS (both tagged and untagged) were also calculated using footage collected from a 7-hour, continuous, unmanned aerial vehicle (drone) video survey of one location amidst a JWS aggregation on 4 September 2020, between 08:00–15:00. The drone was kept stationary using a pre-planned mission via Litchi software (VC technology LTD) and the high-resolution onboard GPS unit in the Phantom 4 Pro V2 quadcopter (Da-Jiang Innovations). To reduce the effect angled observations may have on measurements, the 4k resolution (3840x2160) camera was positioned perpendicular to the ocean surface. This created a static “arena” with which to observe JWS movements within the aggregation. The drone’s field of view was converted to a real distance using multiple, cross-referenced ground sampling distance (gsd) calculators (propellaero.com and handelselaras.com). The gsd calculators provided distance in cm/pixel which was then used to determine distance of the length and width of field of view. Overall, we observed an area of 0.53 km^2^ for 7 hrs. Pixels are calibrated into accurate distances using ground sample distance (gsd) calculators (Propeller Aerobotics Pty, Surry Hills, NSW, AU). By incorporating the focal length, camera sensor dimensions, width of the image, and altitude of the drone, individual pixels within the frame of the drone can be calibrated into a length in meters. Using a stable drone altitude of 60 m, each pixel was equivalent to 0.0278 m. To calculate swim speed, images were taken every second individual sharks were within the frame of the drone, and distance traveled between each frame was measured in pixels. This pixel measurement was then converted to a distance in meters using the gsd. We repeated this process for every shark that passed within the frame of the drone over a 7 hr period. We swapped between two drones observing the same position since drone battery life is ~20 min. Sharks whose path/swim speed measurements were disrupted by a drone swap were not included in this study.

The drone was programmed to stay at a single location and a single height using Litchi Drone Software (VC Technology, London, UK). This software autonomously positions the drone using the high-resolution onboard GPS unit and barometer (0.3 m vertical and horizontal potential error) to limit error derived from human controlled flight.

We observed 97 instances of sharks within the survey arena from 08:00 to 15:00. Duration of time within the arena ranged from 5 to 479 s with a mean of 77 ± 83 s. Shark observations were only used to calculate swim speeds if they were present in the arena for 60 s or more. Five shark observations were processed for every hour of the total 7-hour survey. Two hours had to be excluded, 11:00 to 12:00 PST and 12:00 to 13:00 PST, because sharks were not present in the arena for more than 60 s. Images from the video were extracted every two seconds from when the shark entered the arena to when the shark exited the arena. Distance traveled between time-steps was measured via ImageJ (v. 1.8.0, NIH, MD, USA) by again converting pixels measured to cm/pixel via gsd calculators. This was then converted to m/s. Sharks sizes were measured using this same method. Shark sizes were only measured using frames when the shark was directly below the drone. 1237 swim speeds were calculated with an average of 49 data points per shark across 25 shark observations. Sharks included in these calculations ranged in size from 1.82 m to 2.90 m.

### Field routine metabolic rate (fRMR) and whole body routine metabolic rate calculation

Field RMR was calculated following methods described in Semmens et al. (2013), whereby a swimming performance curve generated for juvenile shortfin mako sharks [23] (closely related species) was combined with the routine oxygen consumption rates slope from juvenile white sharks [26] to derive oxygen consumption rates (MO_2_) from calculated swim speeds of subadult and adult white sharks in their study. Briefly, shark cruising speeds (median SS_*H*_ values) were used to calculate the associated physiological oxygen requirement (oxygen consumption rate), as a proxy for metabolic rate using Eq (1) below:

$$log\left(MO_{2}\right)=0.58\left(U\right)+log\left(246\right)$$ (1)

whereby log(246) represents the empirical standard metabolic rate determined for (captive) young of the year white sharks [26], *U* is the cruise speed (in total-lengths per second) derived from median V_*H*_ value for the shark, and 0.58 is the slope of the curve for the allometric relationship between body mass and oxygen consumption rates for a mako shark, as described by Graham et al. (1990) and applied by Semmens et al. (2013) to mature/adult great white sharks in their study. fRMR was calculated via the median cruising speed for each shark and also as the “grand” median (the mean of all the median fRMR’s).

Mass-specific fRMR was calculated via two different methods. Mass-specific fRMR was derived from for each individual according to general equation supplied by Semmens et al. (2013). Mean mass-specific fRMR based on the mean body size of all JWS included in the study was then derived from the associated plot of MO_2_ vs mass. For comparative purposes we also estimated whole body RMR based upon the details supplied by Payne et al (2015), which used the general equation:

$$aM^{b}$$ (2)

where *a* is the antilog of the intercept of the lamnid curve (from Ezcurra et al. (2012), *M* is log_10_ (body mass) (in kg) and *b* is the allometric scaling exponent (in this case 0.79). Mean whole body RMR using this method was again subsequently derived using the mean body size of all JWS included in the study.

### Block bootstrap hypothesis tests

Many hypothesis tests concern comparing two samples and drawing conclusions about their possibly similarities or differences (e.g. t-tests). Oftentimes, they rely on the assumption of independent sampling. Serial measures of position, velocity, and environmental conditions typically exhibit autocorrelation and fail to satisfy this assumption. This can lead to an underestimation of the sample variance and thus overly optimistic conclusions. The bootstrap (Efron and Tibshirani, 1993) [59] is commonly employed in these difficult situations where traditional methods may be inappropriate. Specific to autocorrelated data, the block-bootstrap (Wilks 1997) [60] is an appropriate alternative to t-tests and similar comparisons of sample means.

In a vanilla bootstrap, the sampling distributing of the desired statistic S is approximated by repeatedly sampling the original data D to create a “new” sample D* and calculating S(D*). After many repetitions, the distribution of the sampled statistic can be used to construct confidence intervals and perform equivalent two-sided hypothesis tests. Block bootstrapping preserves the important autocorrelation in the data by resampling blocks of length L with replacement and appending them to make D*.

Here, we conducted several tests concerning median swim velocities using the block bootstrap. Throughout, we performed 10,000 bootstrap samples using L = 100 to find the distribution of the test statistic; the significance level was α = 0.05 unless otherwise noted. To test whether median swim speeds were shared by female, male, and unidentified sharks, we performed three pairwise comparisons. In order to maintain a familywise error rate of 0.05, we applied the Bonferroni correction and performed these tests at the α = 0.05/3 = 0.01667 level. These tests suggested female sharks had significantly higher median swim speeds than male or unidentified sharks and that male and unidentified sharks did not significantly differ.

### Environmental temperature collection and interpolation

Water temperature values at the sea surface and at receiver depth (0.25 m from the bottom) were collected throughout the duration of the study. Surface temperatures were collected from using either the inbuilt thermistor of a Vemco VR2W tx acoustic receiver (hourly sampling, 0.1°C resolution [Vemco|Innovasea, Nova Scotia, Canada]) or an ElectricBlue Environmental Logger (hourly sampling, 0.1°C resolution [ElectricBlue, Porto, Portugal]). Bottom temperatures were recorded at each receiver location either by the inbuilt thermistors of Vemco VR2W tx acoustic receivers, or by using HOBO Stowaway TidbiT v2 UTBI-001 data loggers (hourly sampling, 0.1°C resolution [Onset Computer Corporation, Bourne, MA]). The temperature dataset was further supplemented with nine vertically undulating periodic surveys of the water column throughout the array, using an Iver 3 autonomous underwater vehicle (L3Harris Technologies | Melbourne, Florida, USA) equipped with a Xylem/YSI sonde suite (YSI Inc. | Yellow Springs, Ohio, USA).

This environmental dataset (752,193 total observations from May to December 2020, with temperatures ranging from 10.9°C to 35.5°C) was then used to train a machine learning algorithm, specifically a Feed Forward Neural Network (FFFN), to model three-dimensionally model water temperature as a function of location, depth, and time throughout the array.

A feed forward neural network composes successive non-linear transformations of input variables to produce its final output. Specifically, an input x is transformed according to

$$f\left(x\right)≔f_{L}\circ f_{L-1}\circ \cdots \circ f_{1}\left(x\right)$$

where each *f*_*l*_ represents a successive transformation. Each transformation, or layer, accepts the output **z** from the previous layer and transforms it according to

$$f_{l}(z)≔h(W_{l}z+b_{l})$$

The parameter matrix *W*_*l*_ and vector **b**_*l*_ produce a linear transformation of the input, and their dimensions may be chosen to suit the given problem. The function *h* is referred to as the “activation function”, and it imparts the non-linearity to the transformation. Throughout we use element-wise ReLU: *h*(*z*): = max(0, *z*). Determining the number of layers, the dimensions of all *W*_*l*_, *b*_*l*_, and estimating their contents constitutes fitting the model.

Our model consists of 11 such transformation layers and a final linear transformation to produce a scalar output. The model receives the input **x** ∈ R5 containing

- latitude, degrees north
- longitude, degrees east
- depth, meters
- day of year
- time of day, minutes

The first layer maps **x** to 128 dimensions i.e.


$$f_{1}\left(x\right)=h\left(W_{1}x+b_{1}\right);\;\mathrm{where}\;W_{1}\in R^{128\times 5},b_{1}\in R^{128}$$

The ten successive layers maintain the output shape of 128. A final layer does not include an activation function and produces a scalar output:

$$f_{L}(z)=W_{L}z+b_{L};\;\mathrm{where}\;W_{L}\in R^{1\times 128},b_{L}\in R$$

We implemented our model in Tensorflow 2.4.1 for Python 3.7.10. We fit our model using stochastic gradient descent with the Adam (*α* = 0.001, *β*_1_ = 0.9, *β*_2_ = 0.999, *ε* = 10^*−*8^) optimizer and batch size of 128 over 40 epochs.

The final training error demonstrates the accuracy of this network, producing a final mode; with mean absolute error rate *<* 0.3°C, about 1% of the range of the response.

## Supporting information

S1 Fig Violin plot of distributions of calculated swim speeds for each shark included in the study, colored according to sex. (DOCX)

S2 Fig Regressions of estimated O_2_ consumption rates against shark body mass. Relationships between O_2_ consumption and body mass are governed by the scaling coefficient used in the general equation log_10_ MO_2_ = log_10_(SMR) + *coeff*.(*U*). Points and lines show values generated according to coefficients of 0.58 (magenta; used by Semmens et al. 2013), 0.79 (green; the allometric scaling used by Ezcurra et al. (2012) and Payne et al (2015), and 0.97 (blue; used by Watanabe et al. 2019).
(DOCX)
