# Supplementary material for: High resolution acoustic telemetry reveals swim speeds and inferred field metabolic rates in juvenile white sharks (Carcharodon carcharias)
Source: PLoS One. 2022 Jun 9;17(6):e0268914. doi: 10.1371/journal.pone.0268914 (PMC9182713; doi:10.1371/journal.pone.0268914)
Supplement: S1 Fig — (DOCX) [file pone.0268914.s001.docx]

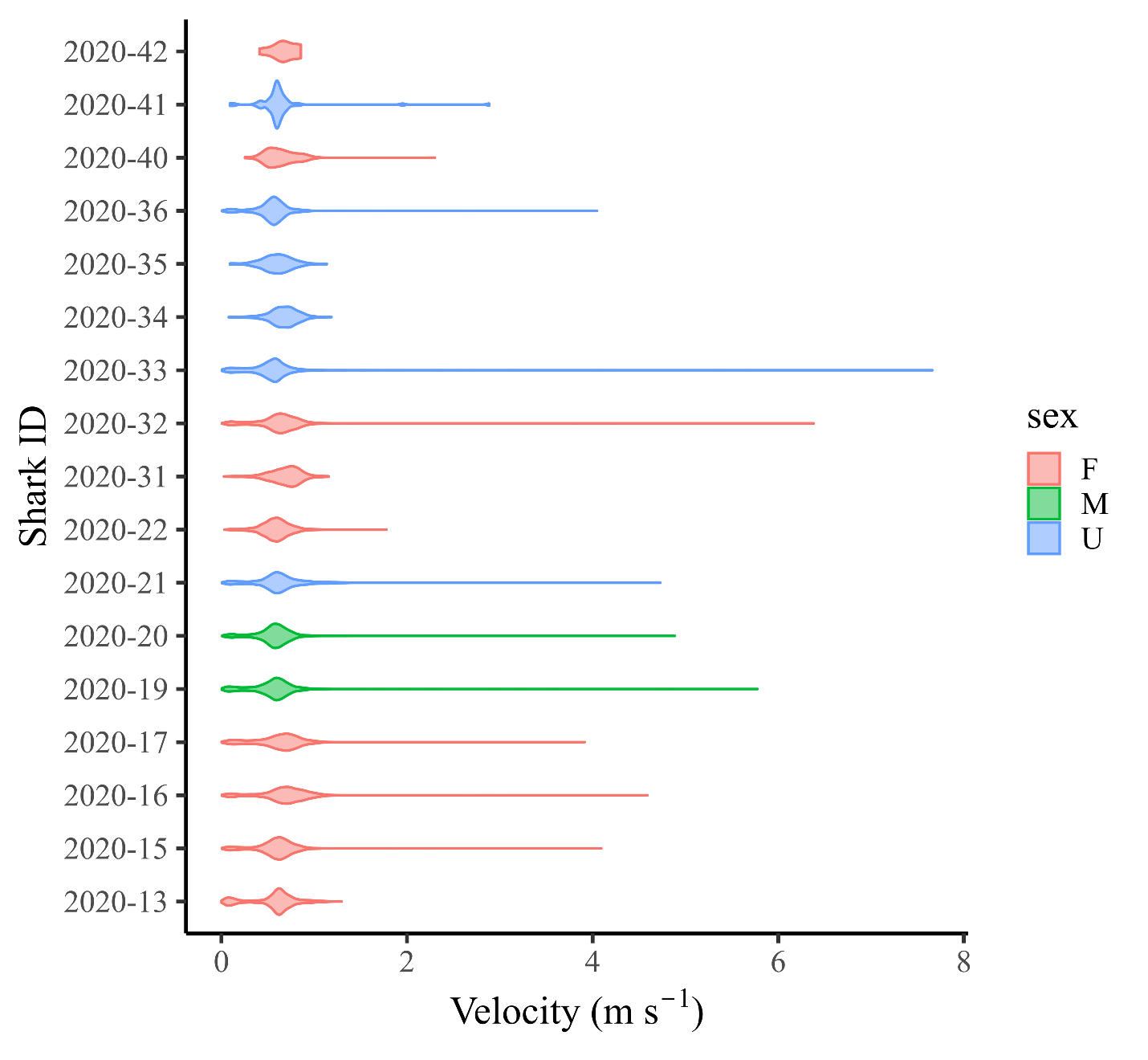


**S1 Fig.** Violin plot of distributions of calculated swim speeds for each shark included in the study, colored according to sex.
