# Supplementary material for: High resolution acoustic telemetry reveals swim speeds and inferred field metabolic rates in juvenile white sharks (Carcharodon carcharias)
Source: PLoS One. 2022 Jun 9;17(6):e0268914. doi: 10.1371/journal.pone.0268914 (PMC9182713; doi:10.1371/journal.pone.0268914)
Supplement: S2 Fig — Relationships between O2 consumption and body mass are governed by the scaling coefficient used in the general equation log10 MO2 = log10(SMR) + coeff.(U). Points and lines show values generated according to coefficients of 0.58 (magenta; used by Semmens et al. 2013), 0.79 (green; the allometric scaling used by Ezcurra et al. (2012) and Payne et al (2015), and 0.97 (blue; used by Watanabe et al. 2019). (DOCX) [file pone.0268914.s002.docx]

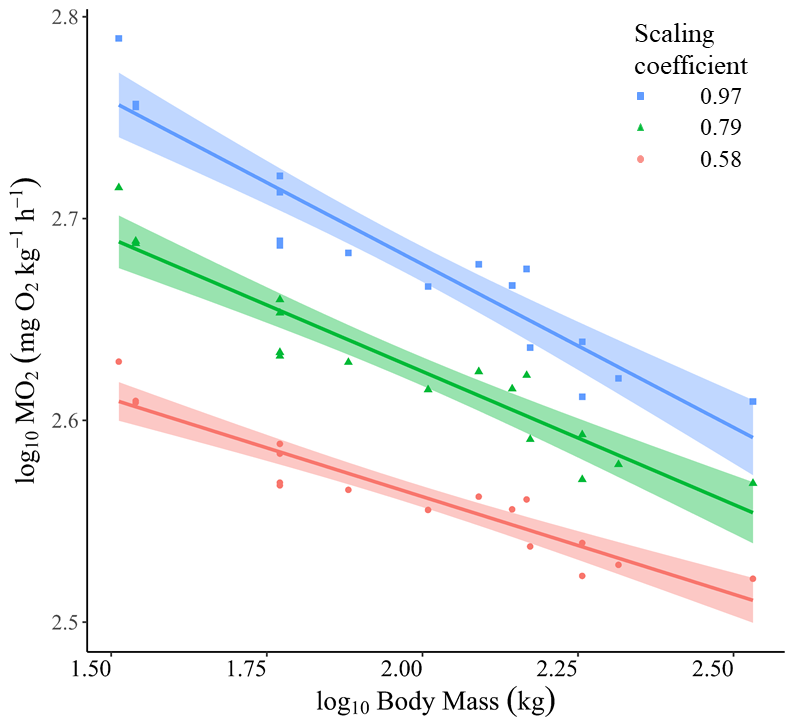


**S2 Fig.** Regressions of estimated O_2_ consumption rates against shark body mass. Relationships between O_2_ consumption and body mass are governed by the scaling coefficient used in the general equation log_10_ MO_2_ = log_10_(SMR) + *coeff.*(*U*) . Points and lines show values generated according to coefficients of 0.58 (magenta; used by Semmens et al. 2013), 0.79 (green; the allometric scaling used by Ezcurra et al. (2012) and Payne et al (2015), and 0.97 (blue; used by Watanabe et al. 2019).
